# Supplementary material for: Task-optimized models of sensory uncertainty reproduce human confidence judgments
Source: bioRxiv. 2025 Nov 2:2025.10.31.685933. Preprint. [Version 1] doi: 10.1101/2025.10.31.685933 (PMC12636618; doi:10.1101/2025.10.31.685933)
Supplement: Supplementary file 1 [file NIHPP2025.10.31.685933v1-supplement-1.pdf]

bioRxiv preprint doi: https://doi.org/10.1101/2025.10.31.685933; this version posted November 2, 2025. The copyright holder for this preprint (which was not certified by peer review) is the author/funder, who has granted bioRxiv a license to display the preprint in perpetuity. It is made available under aCC-BY 4.0 International license.

Supplementary Information for Govindarajan et al.

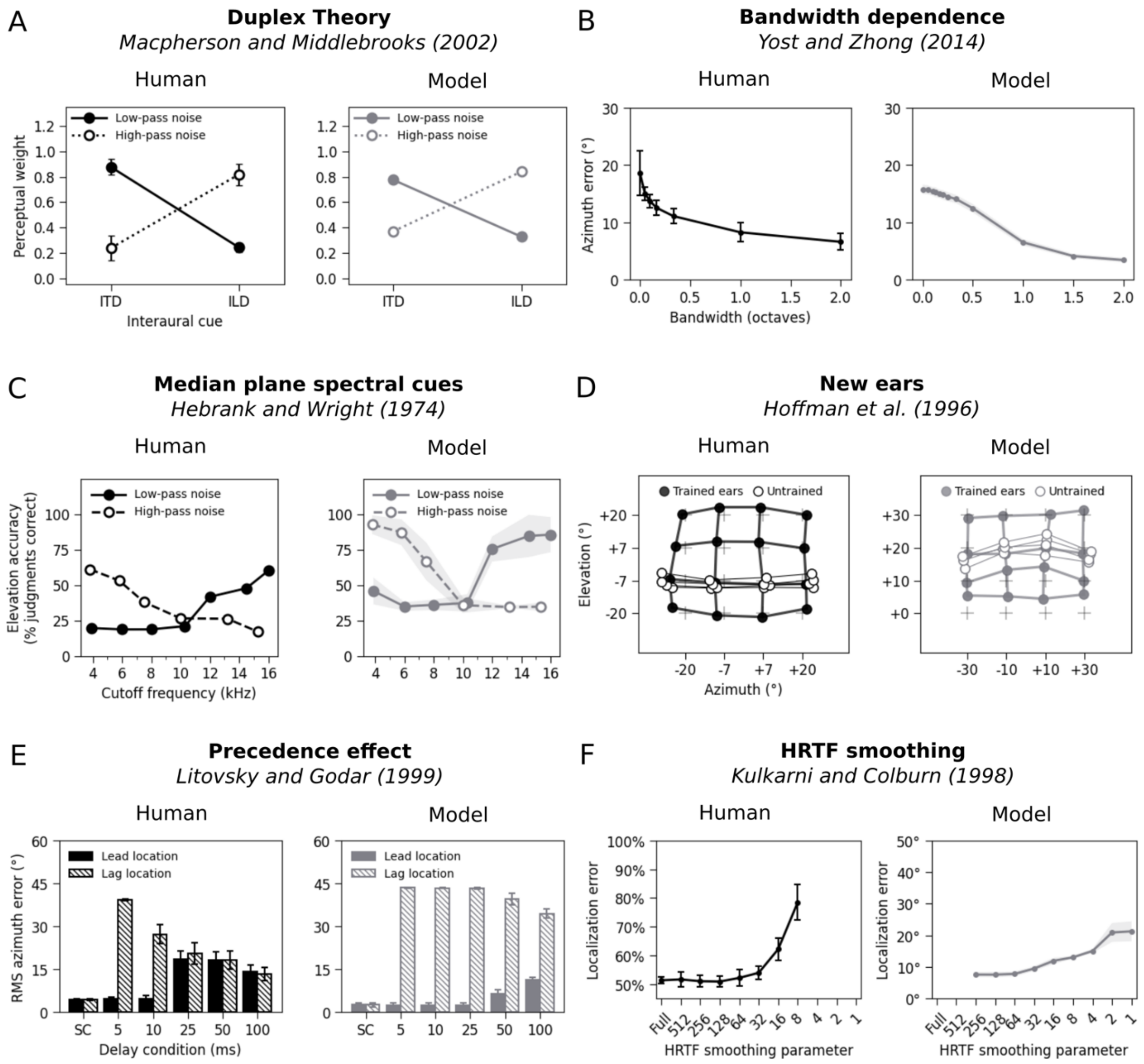

Supplementary Figure 1. Validation of sound localization model. Each panel plots human and model results from the battery of experiments used by Francl and McDermott (2022)[20] for model validation. Each experiment captures an aspect of human spatial hearing. The model qualitatively reproduces each effect.

bioRxiv preprint doi: https://doi.org/10.1101/2025.10.31.685933; this version posted November 2, 2025. The copyright holder for this preprint (which was not certified by peer review) is the author/funder, who has granted bioRxiv a license to display the preprint in perpetuity. It is made available under aCC-BY 4.0 International license.

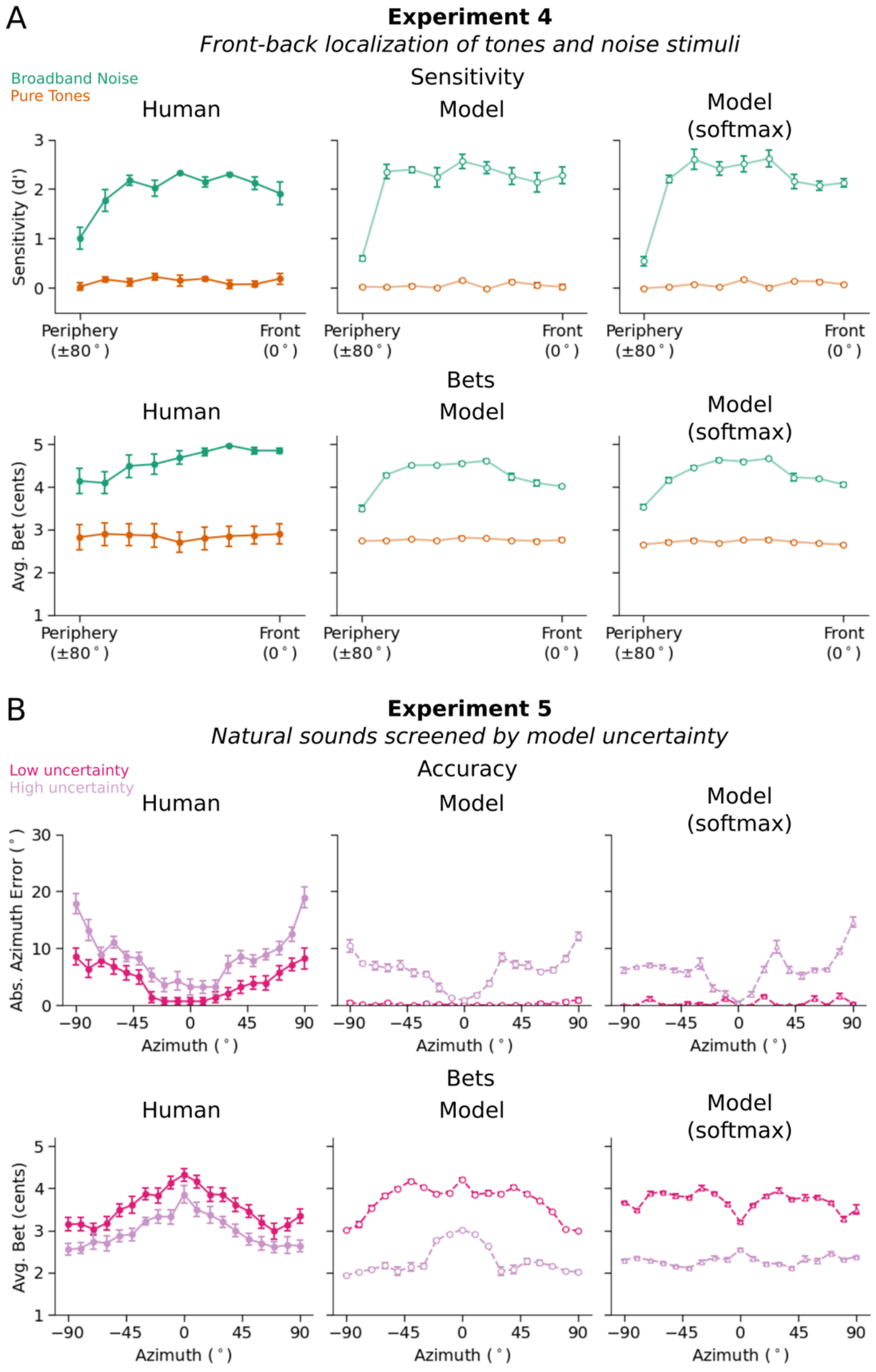

Supplementary Figure 2. Comparison of human-model similarity between an uncertainty-aware model trained with a log-likelihood loss function and a classification model trained with cross-entropy loss. **A.** Experiment 4: Front-back localization of tones and noise stimuli. **B.** Experiment 5: Natural sounds screened by model uncertainty. Error bars plot SEM.

bioRxiv preprint doi: https://doi.org/10.1101/2025.10.31.685933; this version posted November 2, 2025. The copyright holder for this preprint (which was not certified by peer review) is the author/funder, who has granted bioRxiv a license to display the preprint in perpetuity. It is made available under aCC-BY 4.0 International license.

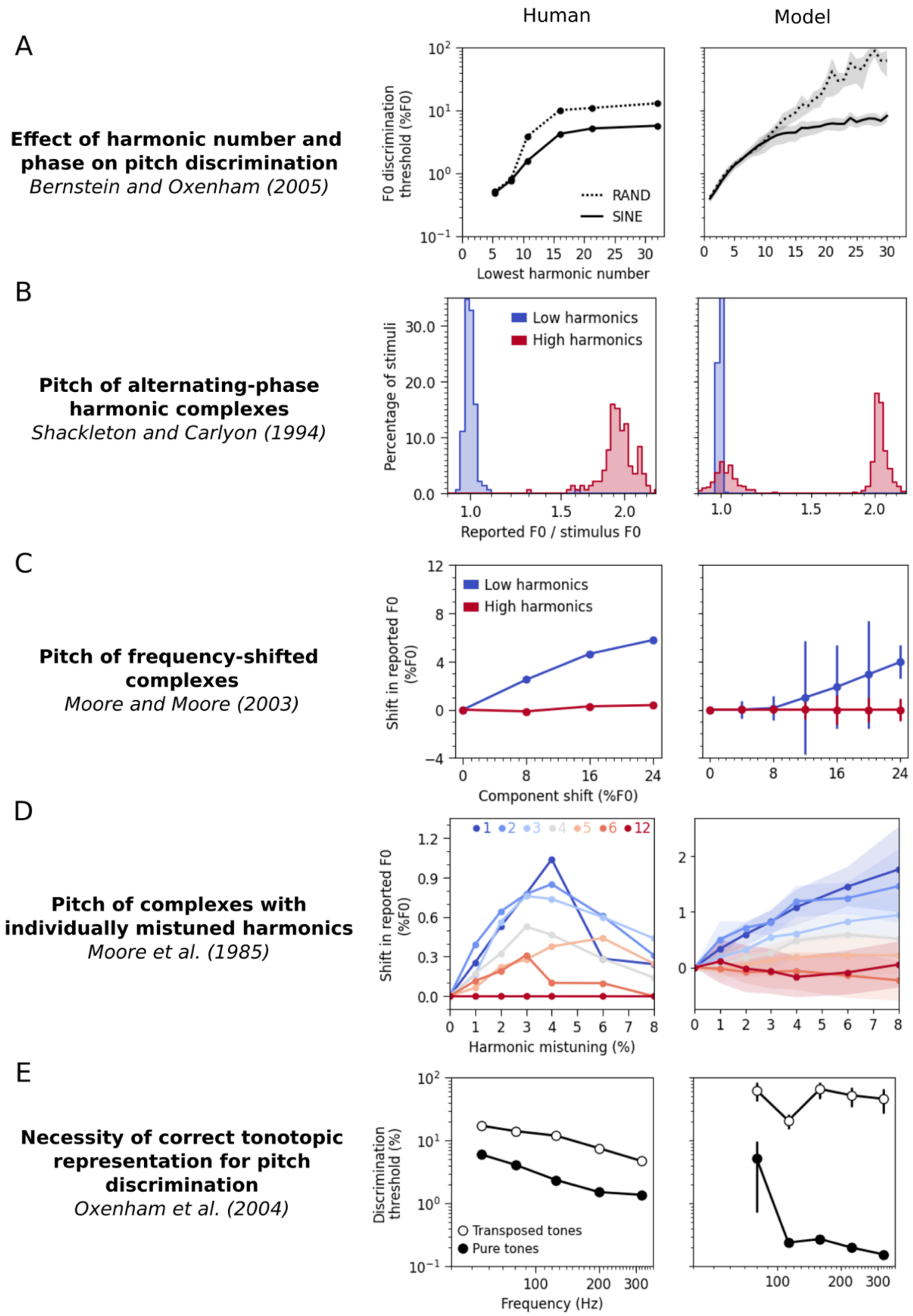

Supplementary Figure 3. Validation of pitch estimation model. Each panel plots human and model results from the battery of experiments used by Saddler et al. (2021)[21] for model validation. Each experiment captures an aspect of human pitch perception. The model qualitatively reproduces each effect.

bioRxiv preprint doi: https://doi.org/10.1101/2025.10.31.685933; this version posted November 2, 2025. The copyright holder for this preprint (which was not certified by peer review) is the author/funder, who has granted bioRxiv a license to display the preprint in perpetuity. It is made available under aCC-BY 4.0 International license.

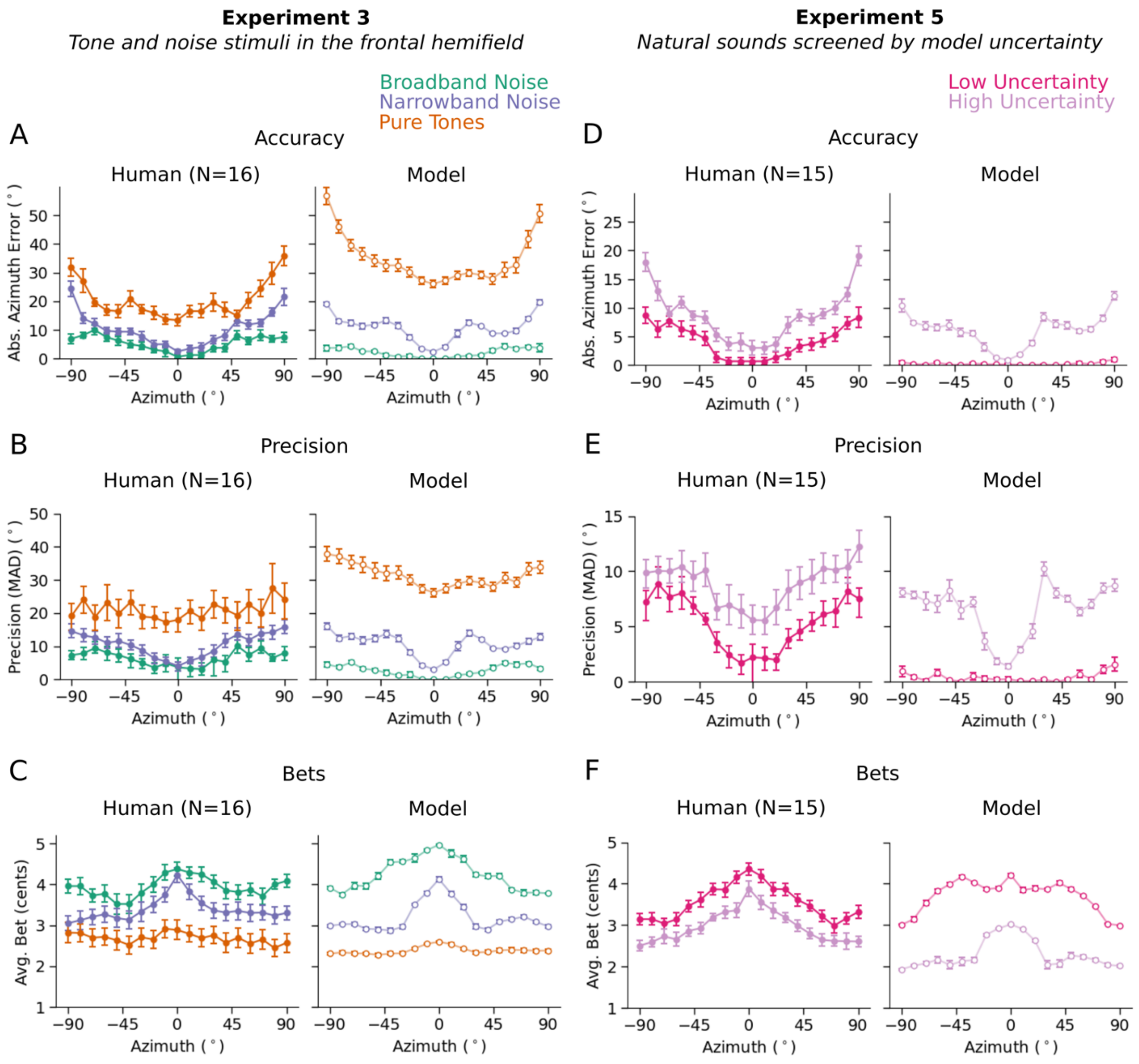

Supplementary Figure 4. Human (left panels) and model (right panels) performance as a function of azimuth for Experiments 3 and 5. **A&D.** Localization accuracy for each stimulus type at each azimuthal position. **B&E.** Localization precision for each stimulus type at each azimuthal position. **C&F.** Bets for each stimulus type and azimuthal position. Error bars plot SEM.

bioRxiv preprint doi: https://doi.org/10.1101/2025.10.31.685933; this version posted November 2, 2025. The copyright holder for this preprint (which was not certified by peer review) is the author/funder, who has granted bioRxiv a license to display the preprint in perpetuity. It is made available under aCC-BY 4.0 International license.

Layer	Type	Input Shape	Output Shape	Params	Details
000_Conv2d	Conv2d	[1, 2, 40, 8000]	[1, 32, 38, 7969]	6144	k=(3, 32), s=(1, 1), p=(0, 0)
001_MaxPool2d	MaxPool2d	[1, 32, 38, 7969]	[1, 32, 38, 7969]	0	k=(1, 1), s=(1, 1)
002_ReLU	ReLU	[1, 32, 38, 7969]	[1, 32, 38, 7969]	0	
003_BatchNorm2d	BatchNorm2d	[1, 32, 38, 7969]	[1, 32, 38, 7969]	64	
004_Conv2d	Conv2d	[1, 32, 38, 7969]	[1, 32, 37, 7954]	32768	k=(2, 16), s=(1, 1), p=(0, 0)
005_MaxPool2d	MaxPool2d	[1, 32, 37, 7954]	[1, 32, 37, 1988]	0	k=(1, 4), s=(1, 4)
006_ReLU	ReLU	[1, 32, 37, 1988]	[1, 32, 37, 1988]	0	
007_BatchNorm2d	BatchNorm2d	[1, 32, 37, 1988]	[1, 32, 37, 1988]	64	
008_Conv2d	Conv2d	[1, 32, 37, 1988]	[1, 64, 36, 1957]	131072	k=(2, 32), s=(1, 1), p=(0, 0)
009_MaxPool2d	MaxPool2d	[1, 64, 36, 1957]	[1, 64, 36, 1957]	0	k=(1, 1), s=(1, 1)
010_ReLU	ReLU	[1, 64, 36, 1957]	[1, 64, 36, 1957]	0	
011_BatchNorm2d	BatchNorm2d	[1, 64, 36, 1957]	[1, 64, 36, 1957]	128	
012_Conv2d	Conv2d	[1, 64, 36, 1957]	[1, 64, 34, 1954]	49152	k=(3, 4), s=(1, 1), p=(0, 0)
013_MaxPool2d	MaxPool2d	[1, 64, 34, 1954]	[1, 64, 34, 488]	0	k=(1, 4), s=(1, 4)
014_ReLU	ReLU	[1, 64, 34, 488]	[1, 64, 34, 488]	0	
015_BatchNorm2d	BatchNorm2d	[1, 64, 34, 488]	[1, 64, 34, 488]	128	
016_Conv2d	Conv2d	[1, 64, 34, 488]	[1, 128, 32, 481]	196608	k=(3, 8), s=(1, 1), p=(0, 0)
017_MaxPool2d	MaxPool2d	[1, 128, 32, 481]	[1, 128, 32, 120]	0	k=(1, 4), s=(1, 4)
018_ReLU	ReLU	[1, 128, 32, 120]	[1, 128, 32, 120]	0	
019_BatchNorm2d	BatchNorm2d	[1, 128, 32, 120]	[1, 128, 32, 120]	256	
020_Conv2d	Conv2d	[1, 128, 32, 120]	[1, 256, 30, 119]	196608	k=(3, 2), s=(1, 1), p=(0, 0)
021_MaxPool2d	MaxPool2d	[1, 256, 30, 119]	[1, 256, 30, 59]	0	k=(1, 2), s=(1, 2)
022_ReLU	ReLU	[1, 256, 30, 59]	[1, 256, 30, 59]	0	
023_BatchNorm2d	BatchNorm2d	[1, 256, 30, 59]	[1, 256, 30, 59]	512	
024_Conv2d	Conv2d	[1, 256, 30, 59]	[1, 512, 29, 52]	2097152	k=(2, 8), s=(1, 1), p=(0, 0)
025_MaxPool2d	MaxPool2d	[1, 512, 29, 52]	[1, 512, 29, 52]	0	k=(1, 1), s=(1, 1)
026_ReLU	ReLU	[1, 512, 29, 52]	[1, 512, 29, 52]	0	
027_BatchNorm2d	BatchNorm2d	[1, 512, 29, 52]	[1, 512, 29, 52]	1024	
028_Conv2d	Conv2d	[1, 512, 29, 52]	[1, 512, 27, 49]	3145728	k=(3, 4), s=(1, 1), p=(0, 0)
029_MaxPool2d	MaxPool2d	[1, 512, 27, 49]	[1, 512, 27, 24]	0	k=(1, 2), s=(1, 2)
030_ReLU	ReLU	[1, 512, 27, 24]	[1, 512, 27, 24]	0	
031_BatchNorm2d	BatchNorm2d	[1, 512, 27, 24]	[1, 512, 27, 24]	1024	
032_Conv2d	Conv2d	[1, 512, 27, 24]	[1, 512, 27, 22]	786432	k=(1, 3), s=(1, 1), p=(0, 0)
033_MaxPool2d	MaxPool2d	[1, 512, 27, 22]	[1, 512, 27, 22]	0	k=(1, 1), s=(1, 1)
034_ReLU	ReLU	[1, 512, 27, 22]	[1, 512, 27, 22]	0	
035_BatchNorm2d	BatchNorm2d	[1, 512, 27, 22]	[1, 512, 27, 22]	1024	
036_Linear	Linear	[1, 304128]	[1, 512]	155714048	in=304128, out=512
037_Linear	Linear	[1, 304128]	[1, 512]	155714048	in=304128, out=512
038_ReLU	ReLU	[1, 512]	[1, 512]	0	
039_BatchNorm1d	BatchNorm1d	[1, 512]	[1, 512]	1024	
040_Dropout	Dropout	[1, 512]	[1, 512]	0	
041_Linear	Linear	[1, 512]	[1, 35]	17955	in=512, out=35
042_Linear	Linear	[1, 512]	[1, 35]	17955	in=512, out=35
043_Softmax	Softmax	[1, 5]	[1, 5]	0	
044_Tanh	Tanh	[1, 20]	[1, 20]	0	
045_ReLUX	ReLUX	[1, 10]	[1, 10]	0	

Supplementary Table 1. Sound localization model architecture.

bioRxiv preprint doi: https://doi.org/10.1101/2025.10.31.685933; this version posted November 2, 2025. The copyright holder for this preprint (which was not certified by peer review) is the author/funder, who has granted bioRxiv a license to display the preprint in perpetuity. It is made available under aCC-BY 4.0 International license.

Layer	Type	Input Shape	Output Shape	Params	Details
000_Conv2d	Conv2d	[1, 1, 100, 1000]	[1, 64, 98, 924]	14784	k=(3, 77), s=(1, 1), p=(0, 0)
001_ReLU	ReLU	[1, 64, 98, 924]	[1, 64, 98, 924]	0	
002_HanningPooling	HanningPooling	[1, 64, 98, 924]	[1, 64, 98, 462]	0	
003_BatchNorm2d	BatchNorm2d	[1, 64, 98, 462]	[1, 64, 98, 462]	128	
004_Conv2d	Conv2d	[1, 64, 98, 462]	[1, 128, 98, 270]	1581056	k=(1, 193), s=(1, 1), p=(0, 0)
005_ReLU	ReLU	[1, 128, 98, 270]	[1, 128, 98, 270]	0	
006_HanningPooling	HanningPooling	[1, 128, 98, 270]	[1, 128, 25, 90]	0	
007_BatchNorm2d	BatchNorm2d	[1, 128, 25, 90]	[1, 128, 25, 90]	256	
008_Conv2d	Conv2d	[1, 128, 25, 90]	[1, 128, 18, 81]	1310720	k=(8, 10), s=(1, 1), p=(0, 0)
009_ReLU	ReLU	[1, 128, 18, 81]	[1, 128, 18, 81]	0	
010_HanningPooling	HanningPooling	[1, 128, 18, 81]	[1, 128, 9, 14]	0	
011_BatchNorm2d	BatchNorm2d	[1, 128, 9, 14]	[1, 128, 9, 14]	256	
012_Conv2d	Conv2d	[1, 128, 9, 14]	[1, 256, 8, 13]	131072	k=(2, 2), s=(1, 1), p=(0, 0)
013_ReLU	ReLU	[1, 256, 8, 13]	[1, 256, 8, 13]	0	
014_HanningPooling	HanningPooling	[1, 256, 8, 13]	[1, 256, 4, 7]	0	
015_BatchNorm2d	BatchNorm2d	[1, 256, 4, 7]	[1, 256, 4, 7]	512	
016_Conv2d	Conv2d	[1, 256, 4, 7]	[1, 512, 3, 7]	262144	k=(2, 1), s=(1, 1), p=(0, 0)
017_ReLU	ReLU	[1, 512, 3, 7]	[1, 512, 3, 7]	0	
018_HanningPooling	HanningPooling	[1, 512, 3, 7]	[1, 512, 3, 7]	0	
019_BatchNorm2d	BatchNorm2d	[1, 512, 3, 7]	[1, 512, 3, 7]	1024	
020_Conv2d	Conv2d	[1, 512, 3, 7]	[1, 1024, 2, 4]	4194304	k=(2, 4), s=(1, 1), p=(0, 0)
021_ReLU	ReLU	[1, 1024, 2, 4]	[1, 1024, 2, 4]	0	
022_HanningPooling	HanningPooling	[1, 1024, 2, 4]	[1, 1024, 2, 4]	0	
023_BatchNorm2d	BatchNorm2d	[1, 1024, 2, 4]	[1, 1024, 2, 4]	2048	
024_Flatten	Flatten	[1, 1024, 2, 4]	[1, 8192]	0	
025_Linear	Linear	[1, 8192]	[1, 256]	2097408	in=8192, out=256
026_ReLU	ReLU	[1, 256]	[1, 256]	0	
027_BatchNorm1d	BatchNorm1d	[1, 256]	[1, 256]	512	
028_Dropout	Dropout	[1, 256]	[1, 256]	0	
029_Linear	Linear	[1, 256]	[1, 15]	3855	in=256, out=15
030_Softmax	Softmax	[1, 5]	[1, 5]	0	
031_ReLUX	ReLUX	[1, 5]	[1, 5]	0	
032_Softplus	Softplus	[1, 5]	[1, 5]	0	

Supplementary Table 2. Pitch estimation model architecture.
